# Dual Surgeon Operating in Reverse Geometry Total Shoulder Replacement: The Learning Curve and Its Effects on Complication Rates

**DOI:** 10.7759/cureus.23337

**Published:** 2022-03-20

**Authors:** Hammad Parwaiz, Robert Whitham, Matthew Flintoftburt, Andrew Tasker, David Woods

**Affiliations:** 1 Trauma and Orthopaedics, Great Western Hospital, Swindon, GBR; 2 Trauma and Orthopaedics, Yeovil District Hospital, Yeovil, GBR

**Keywords:** learning curve, dual surgeon operating, shoulder, girft, getting it right first time

## Abstract

Aims

We analyse the impact of implementing dual surgeon operating for reverse geometry total shoulder replacement (RGTSR) as part of the “Getting It Right First Time” (GIRFT) recommendations in our shoulder and elbow unit, and the learning curve associated with it.

Methods

We performed a retrospective cohort study comparing operative time and complication rates in patients who underwent RGTSR performed by a single consultant surgeon versus two consultant surgeons over a six-year period in a single centre, in addition to an analysing the learning curve over the same period.

Results

A total of 74 RGTSRs were performed over a six-year period: 35 patients had a single surgeon perform their procedure and 39 had dual surgeon operating. Observed complication rates for RGTSR nearly halved following the introduction of dual surgeon operating (22.9% vs 12.8%, p=0.36). The complication rate for the first 37 cases was 9/37 (24.4%) versus 4/37 (10.8%, p=0.22) for the next 37 cases.

Conclusion

The implementation of dual surgeon operating may lead to reduced operative complications, provide cost savings to the hospital and produced several other non-tangible benefits to the surgeons and the department. An observed reduction in complication rates demonstrates the learning curve associated with this procedure.

## Introduction

The “Getting It Right First Time” (GIRFT) report was first published in 2012 [1] and examined the current state of orthopaedics in England in terms of pathways of care, patient experience and outcomes. It set out, amongst other things, to improve the quality of care, reduce complications and thereby save significant sums of money.

Since then, the Department of Health and NHS England have funded a national pilot of the GIRFT project with the aim of improving medical care within the NHS by reducing unwarranted variations. It focused on elective adult orthopaedic and spinal services in England, involving more than 220 hospitals across the country. It examined clinical outcomes, processes, patient pathways, network arrangements and financial impacts. It found significant variations in practice and outcomes [2].

One recommendation was to introduce dual consultant operating for difficult cases.

Reverse geometry total shoulder replacement (RGTSR) is a complex operation, with reported complication rates between 19-68%, higher in revision surgery [3]. Within our upper limb team, we introduced dual surgeon operating for RGTSR. This article is a retrospective cohort study comparing the complication rates and operative time in RGTSR before and after the implementation of dual surgeon operating, as well as assessing the learning curve associated with the procedure.

## Materials and methods

Dual consultant surgeon operating lists for more complex cases, predominantly RGTSR, were implemented from early 2016, occurring approximately twice per month. Prior to August 2014, we had one upper limb consultant with more than 20 years of experience. Following the appointment of a new upper limb consultant in 2014, dual surgeon operating was performed with the senior and junior consultant as described above. Operation data were collected from the hospital’s electronic theatres database, and further patient demographic data were collected through a combination of the electronic patient records and patient’s hardcopy medical file.

Data were collected following RGTSR between January 2012 and December 2017, and before and after the implementation of dual consultant operating in 2016. We included patient age, gender, length of operation and complications. Complications included all cases of infection (superficial or deep), dislocation, intraoperative fracture, significant blood loss, neurovascular injury, haematoma and return to operating theatre for any other cause. The online statistics software QuickCalcs by GraphPad (San Diego, CA) was used for analysis [4]. A two-tailed t-test was calculated for continuous data, and a Fisher’s exact test was used for categorical data.

## Results

**Figure 1:**
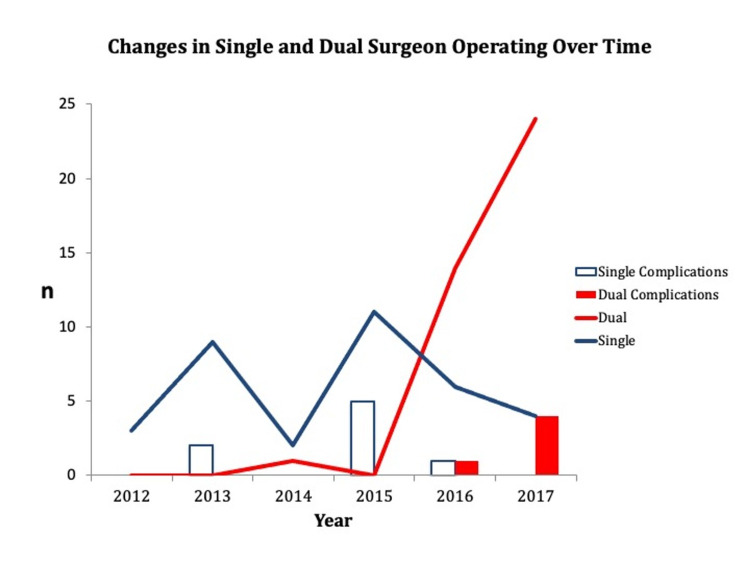
Line graph shows the number of single- and dual-surgeon reverse total shoulder replacements performed in each year from 2012 to 2017. Super-imposed bar charts show the incidence of any complications.

There were 74 RGTSRs performed between January 2012 and November 2017: 35 performed by a single surgeon and 39 by both consultants together (Figure 1). Comparison of the age of the patients, sex, operative time and complications are shown in Table 1.

**Table 1:** A comparison of single versus dual surgeon operating for reverse total shoulder replacements. ^§^Two-tailed t-test. *Two-tailed Fisher’s exact test.

Comparator		Single Surgeon	Dual Surgeon	p-Value
Age (years)	Mean	76.0	75.5	0.80^§^
	Range	54-92	42-87	
Sex	Male	11	14	0.81^*^
	Female	24	25	
Operative time (minutes)		147	144	0.80^§^
Complications		8/35	5/39	0.36^*^

The overall complication rate for single surgeon operating was 8/35 (22.9%) and that for dual surgeon operating was 5/39 (12.8%). The complications in patients operated on by a single surgeon consisted of four post-operative dislocations, one haematoma, one delayed wound healing, one post-operative shoulder stiffness and one hospital-acquired pneumonia. Complications in the dual surgeon operation group included one median/ulnar nerve neurapraxia (resolved after three months), one superficial infection, one dislocation, one acute kidney injury and one early revision of painful prominent metalwork (glenoid base plate screws).

Of the 74 RGTSRs, 64 were primary operations and 10 were revisions. The complication rate for a primary operation was 15.6% (single surgeon 20.7%, dual surgeon 11.4%) and for a revision it was 30% (single surgeon 33.3%, dual surgeon 25.0%). There were fewer observed complications with dual operating but this did not reach statistical significance (p=0.36).

Although there was little difference in the mean operating time between the two groups (Table 1), there was one revision RGTSR in the dual surgeon group which took 256 minutes. This was a difficult operation following a previous open reduction and internal fixation for a proximal humeral fracture. This particular operation lies more than two standard deviations away from the mean (mean: 143; SD: 30.8), and, if excluded as an outlier, the mean operating time for dual surgeon operating would drop to 134 minutes.

When assessing the learning curve of this operation, we examined the complication rates of the first 37 cases performed with the last 37 cases. The complication rate for the first 37 cases was 9/37 (24.4%) versus 4/37 (10.8%, p=0.22) for the next 37 cases, showing a trend towards improvement with experience.

## Discussion

In the wake of the 2015 GIRFT report into orthopaedic services in the UK, there have been changes to practice nationally. Despite this, we are unaware of any published studies on the results of dual operating for RGTSR.

The GIRFT study group has recently published a study protocol for the next phase in their project: to examine whether the planned changes have delivered improvements in the quality of care and patient outcomes [5]. This paper shows evidence of its delivery.

In an era when consultants are being appointed with comparatively less surgical experience compared to yesteryear and surgical juniors are less experienced at assisting, lone operating for a newly appointed consultant carries some clinical and legal risks. There are benefits for the senior surgeon too, as their tried and trusted techniques can be challenged and improved upon by new ideas and methods.

The real and measurable benefits, however, are that dual operating doubles each surgeon’s experience and halves the learning curve. RGTSR has a significant complication rate (24%) and problem rate (44%) [6], particularly when first performed by any individual surgeon, and a lengthy learning curve to reduce this complication rate. Walch et al. reported on two case series from the same surgeon involving 240 cases in each; they brought their complication rate down from 19% to 10.8% [7]. Similarly, Kempton et al. reported on a case series of 200 RGTSRs showing a fall in the complication rate of 23.1% in the first 40 cases to 6.5% in the last 160 cases [8]. The surgeon’s learning curve, and the reduction in complications and reduced operating times associated with it, has similarly been reported by more recent publications, not only in the realm of shoulder surgery [9-13]. We have shown a similar fall in complication rates over time, from 24.4% (first 37 cases) to 10.8% (second 37 cases).

The benefits of dual surgeon operating have been documented extensively in the field of spinal surgery [14-18], but there is paucity of literature on its effects in shoulder surgery, and, specifically, with RGTSR. Dual surgeon operated spinal patients have been shown to have a shorted length of stay [16-17], and in an American spinal study, McDonald et al. showed that the use of two consultant surgeons was associated with a significant increase in the number of cases subsequently performed in the same operating room during business hours [18].

Dual surgeon operating helps with on-table decision-making, and expert assistance means shorter operative time. In our series, the operative time was reducing but had not yet reached statistical significance, perhaps as the surgeons were still on the early part of the learning curve, with a recent systematic review suggesting the learning curve can be variable, with some papers suggesting it takes several hundred cases to reach the plateau phase of the curve [12]. Furthermore, dual surgeon operating reduces the stress of working alone, which has huge benefits with regards to surgeons’ mental welfare and longevity of service.

The limitations of this study are that it was a retrospective cohort study, and the appointment of a second upper limb surgeon in 2014 will have affected the learning curve of the surgeons. It would be interesting to analyse this data prospectively for each individual surgeon, and a larger data set may help to identify if the observed differences reach statistical significance as surgeon experience grows.

Nevertheless, our data add to the body of evidence showing that dual surgeon operating has benefits for patients, surgeons and the health care system through reduction of complications and operative time. This will inevitably bring with it improved patient satisfaction, potentially improved shoulder function, cost savings and more skilled and experienced surgeons.

In addition, implementation of dual surgeon operating comes at a cost to the trust of reduced list capacity overall. The success of our model was dependent on the entire department and hospital managers accepting and implementing the recommendations of our local GIRFT report. We decided to define "difficult cases" as all RGTSRs, but other units may wish to define this differently. With time and increased surgical experience, we may also redefine which cases require dual surgeon operating. Secondly, we had to reconfigure work plans to allow for dual consultant operating on a fortnightly basis. Thirdly, we ran a separate extra list of non-complex cases to compensate for the loss of a consultant-led list. This was performed by one of our associate specialist colleagues. These factors will need to be taken into consideration for any other units wishing to implement such a set-up, but they will also need to be prepared for their own unique challenges based on the environment and trust in which they work.

## Conclusions

Implementing dual surgeon operating for complex cases, as recommended by the GIRFT report, has led to better patient care and a trend towards a decrease in complication rates. It has additional benefits such as enhancing the learning curve, continued professional development of the whole team and a decrease in the stress level of performing difficult cases alone. We believe these simple changes can easily be replicated in other units.
